# Disgust Sensitivity Is Not Associated with Health in a Rural Bangladeshi Sample

**DOI:** 10.1371/journal.pone.0100444

**Published:** 2014-06-30

**Authors:** Mícheál de Barra, M. Sirajul Islam, Val Curtis

**Affiliations:** 1 Environmental Health Group, London School of Hygiene and Tropical Medicine, London, United Kingdom; 2 Environmental Microbiology Lab, ICDDR,B, Dhaka, Bangladesh; University of Stirling, United Kingdom

## Abstract

Disgust can be considered a psychological arm of the immune system that acts to prevent exposure to infectious agents. High disgust sensitivity is associated with greater behavioral avoidance of disease vectors and thus may reduce infection risk. A cross-sectional survey in rural Bangladesh provided no strong support for this hypothesis. In many species, the expression of pathogen- and predator-avoidance mechanisms is contingent on early life exposure to predators and pathogens. Using childhood health data collected in the 1990s, we examined if adults with more infectious diseases in childhood showed greater adult disgust sensitivity: no support for this association was found. Explanations for these null finding and possible directions for future research are discussed.

## Introduction

The emotion disgust is characterized by behavioral avoidance or rejection. A broad range of stimuli including body wastes, deformity, spoilage and certain immoral and sexual acts elicit the emotion [1]. People vary in the degree to which they experience disgust in response to these things, and this variation is known as disgust sensitivity. Disgust often motivates avoidance of things that carry an infection risk; this overlap between infective substances and disgust elicitors suggests that disgust may play a functional role in preventing infection [2]. Over evolutionary timescales the costs associated with parasitism have been an important selection pressure and have sculpted several host defense mechanisms, including behavioral strategies [3]–[5]. Functionally speaking, our feeling of revulsion and the associated avoidance behavior can be considered a psychological arm of the broader immune system [6].

One implication of the parasite-avoidance model is that people prone to strong feelings of disgust will be exposed to pathogens less frequently. Disgust sensitivity is associated with unwillingness to approach/touch things that can cause infection [7] and this reduced exposure could translate into fewer bouts of infectious disease. Consistent with this, Stevenson et al. found that people who were both highly sensitive to disgusting stimuli and inclined to make inferences about spreading contamination reported less recent infections [8]. While the health benefits of disgust were modest, these results suggest that disgust sensitivity can influence health in a high-income population where public health infrastructure is well developed and where infectious disease is rare. In this paper, we examine if individual variation in disgust sensitivity influences infection rates in rural Bangladesh. In this environment, people are exposed to diseases uncommon in high-income settings [9]. If disgust does indeed provide a protective effect, the relationship between infection rate and disgust sensitivity should be clear in this population.

Another prediction derived from the parasite-avoidance model is that disgust sensitivity will be higher where the threat of infection is greater. Systems that protect organisms from pathogens or predators often entail trade-offs: the individual benefits from fewer infections or reduced risk of predation but must pay a cost to develop or maintain the system. The costs of disgust may include rejected food and social partners, or an increased risk of psychopathology [10]. In other species, the costs of disease avoidance can be considerable. For example, Hutchings and colleagues found that sheep with conservative foraging behavior also had lower weight because avoiding pathogens also entailed avoiding high quality forage [11]. Finding the right balance of cost and benefit is an important problem, and one solution lies in facultative expression of the protective system. In humans, food disgust sensitivity appears to decline when the costs of rejection increase, i.e., when people are hungry [12]. Conversely, when the immune system is weakened by pregnancy, there is a compensatory increase in disgust sensitivity [13]. In other species, adjustment to local pathogen/predator risk often occurs in during development and is relatively stable over the lifespan; the organism can use early-life cues to estimate the current threat and develop accordingly [14]. In humans, many life history parameters such as age-at-first birth and age-at-marriage appear to be influenced by early life cues indicative of a risky environment [15], [16]. Local infection risk depends on factors like immunocompetence, sanitation and water infrastructure, local hygiene practices, animal husbandry, and climate. These factors change relatively slowly (i.e., over decades) and so childhood infection rates should provide a reliable medium-term measure of local infection risk and could thus be used to benchmark a locally appropriate level of disgust sensitivity. Consistent with this, we previously found that childhood exposure to disease is associated with a greater preference for opposite sex faces with exaggerated sex typical characteristics, a putative health cue [17]. In other words, people sick more often as children prioritize health cues in partner choice as adults. Thus, our second hypothesis was that people with more infections in childhood would show greater disgust sensitivity in adulthood. We tested this hypothesis using disgust sensitivity data collected in 2010 and longitudinal childhood health data collected in the 1990s.

## Materials and Methods

The data was collected as part of a broader study on health, hygiene and psychology in rural Bangladesh. Participants, all of whom were 16 years of age or older, saw an information sheet, had the aims and methods explained, and were given the opportunity to ask questions. Participants gave written consent before data collection began. Informed consent from participants' next of kin, caretaker or guardian was not sought. The London School of Hygiene and Tropical Medicine Review Board approved the research, including the information sheets, consent forms, and consent procedure.

### Sample

Sample size calculation was based on hypothesis one; that disgust sensitivity will correlate negatively with recent infection frequency. We anticipated that the correlation between disgust and health in the present study would be of small or medium magnitude (r≈.2). A sample size calculation with r = .2, significance threshold  = .05, and power  = .9 indicated that 258 participants were needed.

Participants were randomly selected from a list of people born between July 1990 and August 1997 and living in one of 13 villages familiar to the field workers (i.e., in regions where they had previously conducted field work). The wording of childhood health questions and the frequency of interview remained constant for children born within this period. The sample included 113 men and 171 women and had an average age of 18 years (SD = 1.3). Most participants were unmarried (88%) and Muslim (87%).

### Data collection

Participants were interviewed by one of four FWs (field workers). All four FWs had worked as enumerators in two or more previous research projects. Before data collection began, FWs underwent one week of training that included mock interviews and group discussion. During data collection, progress and evaluation meetings were held every third day. Interviews were conducted in Bengali and took place in the participants' homes in the afternoon or evening. Matlab is the site of a long-term health and demographic research project and people are accustomed to visits from field workers and researchers.

### Measures

The Disgust Scale [18] (revised in [19]) and the Three Domain Disgust Scale [20] are the most commonly used measures of disgust sensitivity. However, neither has been translated into Bengali, and both contain include items with little relevance to a rural, low-income Bangladeshi sample (e.g., “eating vanilla ice cream with ketchup”, “seeing some mold on old leftovers in your refrigerator”). In order to measure disgust sensitivity we designed a simple measure with locally relevant items modeled on these two measures and our previous work on disgust sensitivity in the UK [21]. Consistent with the disease-avoidance model discussed above, these were items related to infectious disease transmission. Hence, this disgust measure focused on what Tybur et al. have termed ‘pathogen disgust’ [20] and what Haidt et al. have referred to as ‘core’ or ‘contagion’ disgust [19]. It is this pathogen related disgust that is most likely to influence participant health and is therefore most relevant to the current analysis. Items were first written in English, then translated into Bengali by a native speaker, and then back translated into English by a second translator who was fluent in both English and Bengali. Minor discrepancies between the versions were resolved through discussion with the translators. Participants were read each item and asked to rate it from 0 (‘not at all disgusting’) to 4 (‘extremely disgusting’). The Bengali and English versions of the questionnaire, as well as the individual-level response data, are available on the Figshare data repository [22].

Participants' current health (i.e. health as adults) was measured using a Benagli- language questionnaire adapted from the 1996 Matlab Health and Socio-Economic Survey [23]. Participants were asked whether or not they had a list of ailments/diseases in the previous twelve months, how many bouts of the illness they experienced, and the recency of the last bout. 73% of participants reported experiencing an infectious disease in the previous 12 months; see Table 1 for more detailed health information. In our analysis *number of infectious diseases* refers to the total number of different infectious diseases experienced in the year before data collection. Following Stevenson et al. [8] we coded the recency of each disease as ‘4’ if the illness was current, ‘3’ if it occurred within the past week, ‘2’ if it occurred in the past month, ‘1’ if it occurred in the past year, and ‘0’ for all other values. By summing the recency score for each disease, an illness recency score was calculated for each participant.

**Table 1:** Illness among the 284 participants in the year prior to data collection.

Disease	Number of participants experiencing disease
Flu	118 (42%)
Gastric Pain	105 (37%)
Cough	82 (29%)
Vomiting	70 (25%)
Diarrhea	61 (21%)
Eye Infection	33 (12%)
Tooth Ache	28 (10%)
Skin Infection	27 (10%)
Fever	9 (3%)
Ear Ache	8 (3%)
Sinus Infection	8 (2%)
Dysentery	7 (0%)
Tuberculosis	0 (%)

The health of participants during their childhood was estimated using data collected by the ICDDR,B in the early nineties [24]. During this period, all mothers of children under 5 years were visited each month and asked if their children had experienced diarrhea in the past fortnight or pneumonia in the past month. These two diseases are the major causes of child mortality in rural Bangladesh [25]. On average, 6.1 (SD: 4.8) bouts of diarrhea and 1.8 (SD: 1.4) bouts were recorded per child. There was a marginal association between childhood diarrhoea and current number of infectious diseases (Spearman's ρ = .11, p = .06) and no association between childhood pneumonia and number of infectious diseases (Spearman's ρ = .06, p = .4). Childhood diarrhea and pneumonia correlate positively (Spearman's ρ = .22, p<.001).

## Results

The raw data and analysis code are available from figshare.com [22]. Disgust responses were first analyzed using exploratory factor analysis. We used the ordinary least squared method to find the solution with minimum residuals. A parallel analysis [26] indicated that the data was best summarized with two factors: The first five eigenvalues were 7.42, 1.76, 1.26, 1.01, and 0.98 while the first three average eigenvalues from 1,000 randomly generated datasets with the same dimensions were 1.59, 1.48, 1.40, 1.33, and 1.29. (95^th^ percentile: 1.68, 1.54, 1.46, 1.38, and 1.31). A scree plot similarly indicated a two-factor solution. For eight of the disgust items, these two latent variables explained less than 20% of the variance and therefore these items were removed and the analysis was repeated [26]. Two items that cross-loaded weakly on both factor 1 and factor 2 were also excluded. To allow for correlation between factors, a direct oblimin rotation was performed. Factor loadings and communality for the surviving items are shown in Table 2. Factor one was primarily associated with unhygienic behavior while several items about people and food loaded on factor two. By averaging the items that loaded most strongly on factor 1 and factor 2 we created two variables, *disgust1* and *disgust2*. These two disgust measures were positively correlated (*r* = .67, *p*<.001). Cronbach's alpha for disgust1 and disgust2 were .85 and .81, respectively.

**Table 2:** Exploratory factor analysis of disgust items: two factor solution.

Back Translated Item	Factor 1	Factor 2	Communality
Picking your nose	.80		.51
Touching the inside of a toilet	.65	.32	.75
Skin with scabies	.59		.35
Eating from dirty plate	.55		.38
Spit on the road	.54		.26
Accidentally using other persons toothbrush	.50		.25
Sour milk	.50		.26
Dead animal	.45		.44
Eating something with left hand	.45		.36
Small acne	.43		.29
Person who never washes himself	.36		.33
Infected eye	.35		.24
Hand without a finger		.79	.64
Deformed body		.67	.56
Perished/decomposed fish		.66	.38
Animal feces in yard		.66	.34
Touching an animal		.53	.52
Eating last nights food		.50	.30
Unkempt beggar		.49	.40
Child with diarrhoea		.38	.23
Very obese man		.31	.22

Note: Items are ordered according to loading on relevant factor. Loadings less than .3 are omitted for clarity.

Men rated disgust1 items (*M* = 3.15 versus 2.96, *t*(282) = 2.74, *p* = .007, Cohen's *d* = .33) and disgust2 items (*M* = 2.62 versus 2.47, *t*(282) = 2.18, *p* = .03, Cohen's *d* = .26) as more disgusting than women did. Disgust sensitivity differed according to which field worker conducted the interview (disgust1 ANOVA: *F*(3,280) = 173, *p*<.001; disgust2: *F*(3, 280) = 149, *p*<.001). These interview effects – i.e., measurement error attributable to characteristics of the interviewer [27] – were accommodated in the analysis using multilevel (mixed) models [28]. Field worker group was modeled as a random effect while disgust, age, and sex were modeled as fixed effects. Using the AIC [29] we compared the fit of different models to the data. In the case of number of infections, a model with no fixed effects (i.e. without disgust, sex or age) fit the data better than a model including disgust, see Table 3. A similar analysis was conducted to investigate the relationship between disgust and recency of infection. As Table 4 indicates, disgust sensitivity had no strong relationship with illness recency.

**Table 3:** A multilevel model of number of infections over past year and disgust sensitivity (two disgust factors).

	Model 1		Model 2		Model 3	
	Estimate	95% CI	Estimate	95% CI	Estimate	95% CI
Fixed effects						
Intercept	2.18	[1.45, 2.91]	3.06	[1.51, 4.63]	2.87	[0.02, 5.75]
disgust1			−0.19	[−0.73, 0.35]	−0.18	[−0.73, 0.36]
disgust2			−0.13	[−0.64, 0.39]	−0.13	[−0.64, 0.40]
sex					0.03	[−0.32, 0.38]
age					0.01	[−0.13, 0.14]
Random effects						
Intercept	0.52		0.39		0.40	
Residual	2.07		2.08		2.10	
Model fit statistics						
Deviance	1023		1022		1022	
Model AIC	1030		1034		1043	
Model AIC – minimum AIC	-		4		13	

**Table 4:** A multilevel model of illness recency and disgust sensitivity (two disgust factors).

	Model 1		Model 2		Model 3	
	Estimate	95% CI	Estimate	95% CI	Estimate	95% CI
Fixed effects						
Intercept	3.32	[2.50, 4.14]	5.28	[2.77, 7.89]	6.33	[0.93, 11.72]
disgust1			−0.51	[−1.48, 0.45]	−0.49	[−1.46, 0.48]
disgust2			−0.17	[−1.11, 0.78]	−0.15	[−1.10, 0.80]
sex					0.21	[−0.48, 0.90]
age					−0.07	[−0.33, 0.19]
Random effects						
Intercept	0.58		0.32		0.32	
Residual	7.99		8.03		8.06	
Model fit statistics						
Deviance	1402		1400		1399	
Model AIC	1408		1410		1416	
Model AIC – minimum AIC	-		2		8	

Childhood health was measured by a different team of FWs and so interviewer effects and the associated correlated error are not relevant in the analysis of childhood health and disgust sensitivity. The data were analyzed using multiple regression. As Table 5 shows, there was no relationship between disgust1 (adjusted *R^2^* = .65, *F*(7,276) = 77.3, *p*<.001) or disgust2 (adjusted *R^2^* = .61, *F*(7,276) = 65.22, *p*<.001) and childhood diarrhea or pneumonia.

**Table 5:** Adult disgust sensitivity and childhood health: multiple regression analyses.

	Disgust1		Disgust2		Disgust: Single factor	
	Beta	95% CI	Beta	95% CI	Beta	95% CI
Intercept	2.64***	[2.06, 3.22]	1.17***	[1.55, 2.79]	2.28***	[1.72, 2.85]
Childhood Diarrhea	0.00	[−0.01, 0.01]	0.00	[−0.01, 0.01]	0.00	[−0.00, 0.01]
Childhood Pneumonia	0.02	[−0.01, 0.05]	0.01	[−0.03, 0.04]	0.02	[−0.01, 0.05]
Field Worker 1 (ref)						
2	0.90***	[0.79, 1.01]	0.31***	[0.20, 0.43]	0.87***	[0.76, 0.98]
3	0.78***	[0.68, 0.90]	1.03***	[0.92, 1.15]	1.26***	[1.16, 1.37]
4	−0.27***	[−0.39, −0.14]	−0.16**	[−0.30, −0.03]	−0.13*	[−0.26, −0.01]
Age	0.00	[−0.03, 0.03]	0.00	[−0.03, 0.04]	0.01	[−0.03, 0.03]
Sex (female)	−0.11*	[−0.20, −0.04]	−0.11*	[−0.20, −0.02]	−0.10**	[−0.18, −0.02]

Note: *** indicates *p*<.001, ** indicates *p*<.01 and * indicates *p*<.05.

Although the parallel analysis and scree plot indicated that disgust sensitivity is best measured by two variables, disgust1 and disgust2, these two factors do correlate strongly and have some overlap in content (e.g., items that could be considered hygiene related load on both factors). Thus disgust sensitivity might be better measured as a single variable. Following a reviewer's recommendation, we reexamined the relationship between disgust sensitivity and health with a single disgust variable. The factor analysis was repeated, items with a communality <.2 were excluded, and all items with a loading of .5 or higher that factor were averaged to created a general disgust score (Cronbach's alpha  = .9; see Table 6). The multivariate analyses results were broadly consistent with those presented above. Number of infectious diseases was best predicted by a simple model excluding disgust (see Table 7). Results displayed in Table 8 indicate that people higher in disgust sensitivity were sick less recently than people lower in disgust. However, this effect was not statistically significant and the AIC statistic indicates that a model excluding disgust provides a better fit. Finally, we found no relationship between disgust and childhood diarrhea or pneumonia (Table 5). To summarize, disgust, when measured as a single construct, is unrelated to the number of infections in childhood or adulthood, or the recency of disease.

**Table 6:** Exploratory factor analysis of disgust items: one factor solution.

Back Translated Item	Factor Loading	Communality
Touching the inside of a toilet	0.83	0.68
A stranger touching your things	0.74	0.55
Eating dropped sweet	0.73	0.53
Touching an animal	0.72	0.52
Deformed body	0.69	0.47
Hand without a finger	0.68	0.46
Dead animal	0.68	0.46
Eating something with left hand	0.62	0.38
Unkempt beggar	0.6	0.36
Person who never washes himself	0.59	0.35
Eating from dirty plate	0.57	0.33
Picking your nose	0.53	0.29
Eating last nights food	0.52	0.27
Sour milk	0.51	0.26

Note: Items ordered according to factor loading

**Table 7:** A multilevel model of number of infections over past year and disgust sensitivity (single disgust factor).

	Model 1		Model 2		Model 3	
	Estimate	95% CI	Estimate	95% CI	Estimate	95% CI
Fixed effects						
Intercept	2.18	[1.45, 2.91]	2.78	[1.41, 4.16]	2.60	[0.20, 5.40]
disgust			−0.22	[−0.68, 0.23]	−0.21	[−0.68, 0.24]
sex					0.04	[−0.31, 0.40]
age					0.01	[−0.13, 0.14]
Random effects						
Intercept	0.52		0.39		0.37	
Residual	2.07		2.08		2.09	
Model fit statistics						
Deviance	1023		1022		1022	
Model AIC	1030		1032		1040	
Model AIC – minimum AIC	-		2		10	

**Table 8:** A multilevel model of illness recency and disgust sensitivity (single disgust factor).

	Model 1		Model 2		Model 3	
	Estimate	95% CI	Estimate	95% CI	Estimate	95% CI
Fixed effects						
Intercept	3.32	[2.50, 4.14]	5.00	[3.01, 6.98]	6.14	[0.96, 11.31]
disgust			−0.62	[−1.32, 0.08]	−0.60	[−1.31, 0.11]
sex					0.23	[−0.46, 0.92]
age					−0.07	[−0.33, 0.19]
Random effects						
Intercept	0.58		0.23		0.22	
Residual	7.99		8.00		8.04	
Model fit statistics						
Deviance	1402		1400		1399	
Model AIC	1408		1409		1414	
Model AIC – minimum AIC	-		1		6	

## Discussion

Contrary to our hypotheses, we found no relationship between disgust sensitivity and childhood health, or between disgust sensitivity and recent health. Below we discuss theoretical and methodological explanations for these null findings

One possible explanation is that disgust sensitivity is unrelated to infection risk. A number of different processes, enumerated below, may weaken the association between disgust and health. (1) Disease exposure depends on community and family behavior as well as individual behavior. If the role of these other people is relatively strong, we are unlikely to detect and relationship between individual psychology and health. Most of our participants were young adults still living at home and their health may therefore be more dependent on parents' and siblings' precautionary behavior. (2) Removing pathogen risks from the one's environment often involves interaction with disgust elicitors and in some circumstances high disgust sensitivity may inhibit actions that benefit health. (3) There is good evidence that people socially learn what constitutes a disgust elicitor [30]. It may be the case that the overlap of disgust elicitors and disease risks is a more important determinant of health than disgust sensitivity itself. In other words, high disgust sensitivity may prevent infection only in individuals who are disgusted by the locally important disease threats. (4) As the findings of Stevenson *et al.* [8] suggests, the interaction of disgust and the tendency to make strong inferences about the spread of contamination may be more important than disgust sensitivity alone. (5) Stevenson *et al.* also suggest that disease exposure results in an increase in disgust sensitivity. Such an effect may mask the protective effects of disgust in a cross-sectional study. Longitudinal data on both disgust sensitivity and health would help to resolve this question.

Our results do not support the hypothesis that adult disgust sensitivity is calibrated by childhood disease exposure. We estimated childhood health using incidence of diarrhea and pneumonia. Although these are important causes of childhood mortality, they kill relatively few adults. Moreover, data from this sample suggests a weak relationship between diarrhea and adult health, and no relationship between pneumonia and adult health. It may be the case the there are other diseases/pathogens that are better predictors of adult pathogen stress and consequently adult disease avoidance behavior.

A more general point is that in some circumstances, a more risky environment can counter-intuitively favor individuals who invest *less* in precautionary behavior [31]. E.g., consider how a solider likely to die in battle gains little in life expectancy from not smoking compared to a general who can expect to survive the war. Unavoidable risks make precautionary behavior for avoidable risks less worthwhile. We have assumed that disease risk is, by in large, an avoidable risk which can be mitigated through precautionary behavior. If, however, a large proportion of infection risk is unavoidable, then individuals in high-risk environments are unlikely to invest more. More research on the extent to which disease risks are avoidable – and are *perceived* to be avoidable – would be help to clarify this issue.

An alternative explanation for these null results is that the measures of health and/or disgust were lacking in validity or reliability. Measuring psychological constructs like disgust sensitivity in a low-income, non-English speaking population is not straightforward. Although Bengali is the 6th most commonly spoken language, few, if any, psychological measures have been translated and validated in Bengali. Our disgust measure followed the format of some commonly used and well-validated measures and was well understood by participants and field workers. However, there are two causes for concern. Contrary to several published studies [2], [32], [33], male participants rated the items more disgusting that women. This indicates that either sex differences in pathogen related disgust are not as consistent as previously argued, or that the measure is biased in some way. However, there are some reasons to think that sex differences in disgust play out differently in this population. In Matlab there is an uneven exposure to disgust-relevant stimuli; women do almost all cleaning and cooking, and they care for infants and the infirm. Rozin has argued that repeated interaction with disgust cues reduces sensitivity [34] and thus this may account for the reversal of sex differences. Another point of concern with the disgust measure was that sensitivity appeared to vary according to field worker. Some interviewer effects are inevitable in this kind of study, and the effects were statistically controlled for in the analysis, but nevertheless they may have weakened our ability to detect a real relationship. Future research on disgust sensitivity may benefit from asking participants to rate pre-recorded audio versions of the items or to rate images of disgust stimuli instead. Another possible explanation for this null result is health was not accurately measured. In particular, our measure of recent health was relatively crude; participants may have forgotten disease events or misremembered the exact timing. However, the positive correlation between childhood and adult disease frequency does suggest these measures have some validity.

We argued that high disgust sensitivity would be associated with fewer infections because more sensitive individuals have less contact with infectious matter. However, actual sickness is a relatively poor measure of pathogen exposure. Most exposure events (e.g., eating contaminated food, being coughed upon) do not result in disease because the pathogenic organisms are destroyed by the immune system or because the infection remains 'latent' [35]. Moreover, people differ in the extent to which they can prevent disease occurring, given exposure. A more direct way to study the protective effects of disgust may be to examine immunological markers of disease exposure. By measuring specific antibody levels, researchers may be able to estimate the frequency of pathogen exposure more accurately [36], [37]. Such a study should have a more power to detect the relationship between disgust and pathogen exposure, if one does indeed exist.
